# Theranostic potential of self-luminescent branched polyethyleneimine-coated superparamagnetic iron oxide nanoparticles

**DOI:** 10.3762/bjnano.13.6

**Published:** 2022-01-18

**Authors:** Rouhollah Khodadust, Ozlem Unal, Havva Yagci Acar

**Affiliations:** 1 Koc University, Department of Chemistry, Surface Science and Technology Center (KUYTAM), Rumelifeneri Yolu, Sariyer, Istanbul, Turkey; 2 University of Health Science, Health Science Institute, Department of Biotechnology Selimiye Mahallesi, Tıbbiye, Uskudar, Istanbul, Turkey; 3 Koc University, Graduate School of Materials Science and Engineering, Rumelifeneri Yolu, Sariyer, Istanbul, Turkey

**Keywords:** Erbitux, photoluminescence, polyethyleneimine, polyinosinic–polycytidylic acid sodium, superparamagnetic iron oxide nanoparticles

## Abstract

Polyethylenimine (PEI), which is frequently used for polyplex formation and effective gene transfection, is rarely recognized as a luminescent polymer. Therefore, it is usually tagged with an organic fluorophore to be optically tracked. Recently, we developed branched PEI (bPEI) superparamagnetic iron oxide nanoparticles (SPION@bPEI) with blue luminescence 1200 times stronger than that of bPEI without a traditional fluorophore, due to partial PEI oxidation during the synthesis. Here, we demonstrate in vitro dye-free optical imaging and successful gene transfection with luminescent SPION@bPEI, which was further modified for receptor-mediated delivery of the cargo selectively to cancer cell lines overexpressing the epidermal growth factor receptor (EGFR). Pro-apoptotic polyinosinic–polycytidylic acid sodium (PIC) was delivered to HeLa cells with SPION@bPEI and caused a dramatic reduction in the cell viability at otherwise non-toxic nanoparticle concentrations, proving that bPEI coating is still an effective component for the delivery of an anionic cargo. Besides, a strong intracellular optical signal supports the optically traceable nature of these nanoparticles. SPION@bPEI nanoparticles were further conjugated with Erbitux (Erb), which is an anti-EGFR antibody for targeting EGFR-overexpressing cancer cell lines. SPION@bPEI-Erb was used for the delivery of a GFP plasmid wherein the transfection was confirmed by the luminescence of the expressed gene within the transfected cells. Poor GFP expression in MCF7, a slightly better expression in HeLa, and a significant enhancement in the transfection of HCT116 cells proved a selective uptake and hence the targeting ability of Erb-tagged nanoparticles. Altogether, this study proves luminescent, cationic, and small SPION@bPEI nanoparticles as strong candidates for imaging and gene therapy.

## Introduction

Luminescent materials are of great interest in biotechnology and medicine since they can be utilized in sensors, labelling, and imaging [1–5]. Luminescent proteins, luminescent synthetic polymers, and quantum dots are the most popular luminescent materials with advantages and disadvantages [6–10]. Luminescent polymers are either π-conjugated systems with delocalized electrons or polymers with conjugated fluorophores as pendant or end groups [11–13]. Conjugated polymers usually suffer from insolubility, which limits their processability and hence medical use [13–14]. However, in recent years, amine-containing branched polymers such as polyethyleneimine (PEI) [15–19] and dendrimers such as polyamidoamine (PAMAM) [20–24] are reported as weakly blue luminescing organic materials. The exact reason behind this luminescence is not known, but it seems like there is not a single mechanism that explains the intrinsic fluorescence of these “periodically amine” containing species. Different factors including delocalization of nonbonding electrons in highly repeating systems, the rigidity of the backbone, acidification of amines, hydrogen bonding, exciplex formation, amine oxidation, and solvent-induced aggregation were reported as factors that amplify the weak luminescence of PEI and amine-containing dendrimers [15–18,20,24]. The luminescence of these materials is especially valuable since they are widely used for drug and gene delivery and may provide “label-free tracking” of these agents in vivo and in vitro [25–26]. Although both PEI and PAMAM have been studied for drug/gene delivery for decades, these systems have not been recognized as luminescent delivery vehicles until recently due to a very weak luminescence. Lin et al. synthesized mostly linear PEI and demonstrated a siRNA delivery with this luminescent polymer [27]. Sun et al. utilized the luminescence of branched PEI as the imaging modality in polymeric quantum dots (PDOTs) formed from amphiphilic polyethyleneimine-polylactide (PEI–PLA). An enhanced luminescence of PEI in the PDOT (quantum yield = 0.31) compared to free PEI (quantum yield = 0.01) was attributed to the more compact structure of PEI in the self-assembled PDOT. This was the first report that studied the luminescence of branched PEI at a relatively high molecular weight (25 kDa) [19].

Polyethyleneimine, especially branched 25 kDa PEI, has been accepted as the golden standard for non-viral nucleic acid delivery, providing efficient binding to the cell surface, endosomal release of the cargo, and translocation to the nucleus [25,28–30]. To develop theranostic nanomaterials, PAMAM and PEI were frequently coupled with superparamagnetic iron oxide nanoparticles (SPIONs) for drug/gene delivery combined with magnetic resonance imaging [31–32]. Usually, these systems were conjugated with other fluorescent tags for optical detection of nanoparticles in cells in many in vitro studies including, for example, flow cytometry or fluorescence imaging, since the luminescence of the polymer was not detected [18,33–34]. Unfortunately, the luminescence of the fluorophores (dye or quantum dots) that are active in the visible range is usually significantly reduced when attached to the iron oxide surface since SPIONs have strong absorption in the UV and visible range of the spectrum [33]. Alternatively, PEI-bound luminescent nanoparticles, such as quantum dots or graphene nanoparticles, are also being studied to combine optical imaging and gene transfection abilities in a single composition.

Recently, we did report an exceptionally strong blue luminesce of branched PEI- (25 kDa) coated SPION nanoparticles [35]. Such strong luminescence in the visible range is very interesting considering that the fluorophore, which is branched PEI (bPEI), is directly attached to SPION which has strong absorbance in the visible window of the electromagnetic spectrum. A tremendous enhancement in the poor, mostly unrecognized and unutilized blue luminescence of bPEI was achieved when it was used as a coating on SPION crystals. We suggested that the partial oxidation of the amines during the synthesis of SPION@bPEI is responsible for the enhanced bPEI luminescence. It is further enhanced with the immobilization of bPEI on SPION crystals and post-synthetic acidification of the particles, which also increased the rigidity. These nanoparticles have a small hydrodynamic size and a positive surface charge. The former is very important for the pharmacokinetics of nanoparticles and needed for long blood circulation time, especially when a molecular targeting is aimed [36–38]. The latter is essential for the highly popular gene therapy, especially in the treatment of cancer [39–40]. Besides, SPIONs are already in the clinic as magnetic resonance imaging (MRI) agents and SPION@bPEI nanoparticles have a strong T2 signal (the signal that reflects the length of time it takes for the MR signal to decay in the transverse plane) [35].

In recent years, there has been a growing demand for a combination of different imaging modalities to improve the detection limit and to provide image-guided therapies [41–42]. Both MRI and optical imaging are noninvasive imaging modalities. Magnetic resonance imaging provides high spatial resolution but lacks sensitivity. Optical imaging, on the other hand, has better sensitivity but suffers from limitations in the penetration depth in in vivo studies. However, it is quite successful in the preclinical research [43]. Hence, a combination of the two modalities provides many advantages. One of the approaches towards such structures are SPIONs conjugated with luminescent quantum dots (QD) [44–47] or tagged with luminescent dyes such as indocyanine green (ICG) [48].

Here, we demonstrate the utility of intensely blue-luminescent, small, and cationic SPION@bPEI in dye-free optical detection and therapeutic gene transfection as well as its targeted delivery to epidermal growth factor receptor (EGFR)-positive cancer cell lines, in vitro. Initially, the dose dependent cytotoxicity of the nanoparticles was determined. Then, using a fluorescence microscope, the ability of these nanoparticles to generate intracellular optical signal was demonstrated. Then, a pro-apoptotic oligonucleotide (polyinosinic–polycytidylic acid sodium salt) (PIC) was transfected into HeLa cells to demonstrate that luminescent bPEI coating is still an effective component for the delivery of an anionic cargo and it may deliver oligonucleotides in a therapeutic dose. Next, SPION@bPEI were conjugated with Erbitux (Erb), which is an anti-EGFR antibody for targeting EGFR-overexpressing cancer cell lines. Finally, SPION@bPEI-Erb nanoparticles were used for the targeted delivery of a GFP plasmid, whose transfection can be confirmed with the luminescence of the expressed gene within the transfected cells. Overall, we have demonstrated the label-free optical tracking, gene transfection, and receptor targeting ability of SPION@bPEI, which exploited its small size, cationic nature and intrinsic luminescence.

## Materials and Methods

### Synthesis of branched PEI-coated SPIONs (SPION@bPEI)

The procedure detailed in our previous study was followed [35]. Briefly, an aqueous reaction mixture composed of 0.07 M of FeCl_2_·4H_2_O (Merck, USA), 0.14 M of FeCl_3_·6H_2_O (Merck, USA), and 0.6 mM of bPEI (Aldrich, USA) was treated with ammonium hydroxide (Sigma-Aldrich, USA) at 80 °C under argon atmosphere. The black solution was cooled to room temperature after 30 min and acidified to pH 5 with CH_3_COOH (Lachema, Czech Republic). The final product SPION@bPEI was washed with DI water using 30 kDa Amicon centrifugal filters and stored at room temperature. The total organic content of the particles was determined by thermogravimetric analysis (TGA).

### Erbitux conjugation to SPION@bPEI

Before any application, Erbitux (Erb) (Merck Serono, UK) with a stock concentration of 5 mg/mL was washed with phosphate-buffered saline (PBS) (Biomatik, Canada) using an ultracentrifugation filter (10 kDa MWCO Amicon). After washing, the concentration of Erb was calculated to be 3 mg/mL using the Bradford assay. An amount of 500 µg of Erb was activated by mixing it with 15 mg of 1-ethyl-3-[3-dimethylaminopropyl]carbodiimide hydrochloride (EDC) (Sigma-Aldrich, USA) and 15.5 mg of *N*-hydroxysulfosuccinimide (sulfo-NHS) (Sigma-Aldrich, USA) in 2-(*N*-morpholino)ethanesulfonic acid (MES) buffer (Biomatik, Canada) at pH 6.0 at room temperature for 15 min. Then, NHS-activated Erb (152 kDa) was washed with PBS (Biomatik, Canada) using an ultracentrifugation filter (10 kDa MWCO Amicon) and then added to 120 mg of SPION@bPEI in PBS. After 48 h of mixing at +4 °C, the reaction was quenched with an excess of hydroxylamine and the product was purified by dialysis in PBS using a 300 kDa dialysis device (Float-A-Lyzer, Spectrum labs, USA) at +4 °C with four times buffer refreshment in 12 h to remove unbound Erb.

The amount of Erb conjugated to nanoparticles was quantified by the Bradford assay. The unbound Erb removed by dialysis was then concentrated. This solution (1 mL) was mixed with 1 mL of the Bradford Reagent (Sigma-Aldrich, USA) for 10 min at room temperature, and then its absorbance at 595 nm was recorded using a UV–vis spectrophotometer (Shimadzu UV-3600 UV–vis–NIR spectrophotometer). A calibration curve was prepared with bovine serum albumin (BSA) at concentration values of 0, 1, 2, 4, 6, 8, and 10 µg/mL in PBS to enable a correlation between the measured absorbance and protein concentration. Then, the concentration of unbound Erb was calculated from its absorbance at 595 nm using the BSA calibration curve. The concentration of bound Erb was calculated by subtracting the unbound amount from the Erb added to the nanoparticles during synthesis. According to this, SPION@bPEI-Erb nanoparticles (12.5 mg/mL) contain 380 µg Erb/mL.

### PIC and pGFP loading to luminescent magnetic nanoparticles (SPION@bPEI/PIC or SPION@bPEI/pGFP)

Polyinosinic–polycytidylic acid sodium salt (Sigma-Aldrich, USA) was dissolved in nuclease-free water to a final concentration of 10 mg/mL. In order to make double-stranded PIC, this solution was heated to 55 °C then cooled back to room temperature according to the instructions from the manufacturer.

As DNA plasmids, GFP plasmids (16542: pBI-MCS-EGFP) were purchased from Addgen, propagated in DH5α competent *E. coli* bacteria, and purified using the QIAGEN EndoFree Plasmid Maxi kit. For this purpose, first the bacteria were cultured in Luria–Bertani agar for single colony selection. The obtained colonies were then grown in Luria–Bertani broth growth medium. Later, the GFP plasmid DNA was isolated from these cells using the QIAGEN Plasmid DNA isolation kit and quantified by the absorbance at 260 nm using the Nanodrop.

Different volumes of PIC or plasmid (500 µg/mL) and SPION@bPEI (5 mg/mL) in HEPES-buffered glucose (HBG, 20 mM of HEPES, 5% w/v of glucose) pH 7.4 were mixed at a final volume of 250 µL and incubated at room temperature for 30 min to generate a series of SPION@bPEI/PIC or SPION@bPEI/pGFP complexes with different N/P ratios. For this purpose, 40 µL of PIC or plasmid (500 µg/mL) was mixed with 2, 4, 8, 16, 32, and 64 µL of nanoparticles (5 mg/mL) which corresponds to N/P ratios of 1.4/1, 2.8/1, 5.6/1, 11.2/1, 22.4/1, and 45/1, respectively. The volume of all samples was completed to 250 µL with HBG. In the N/P calculation, 3 nmol of P per µg of dRNA or dDNA and 10 nmol of N per 0.9 µg of bPEI (25 kDa) were assumed [49].

The loading efficiency was checked by gel electrophoresis. Typically, 1.2 g of agarose was dissolved in 100 mL of tris-borate-EDTA (TBE) (Alfa Aesar, USA) buffer and boiled at 100 °C. After cooled down to 70 °C, 4 µL of ethidium bromide (10 mg/mL) was added and the gel was poured into a gel electrophoresis unit. A volume of 50 µL of the solutions containing 4 µg of PIC were mixed with 5 µL of a 6× DNA gel-loading buffer solution, added into the gel wells, and separated under an electric field (80 mV, 400 mA) for 60 min using a Bio-Rad Mini-Sub Cell GT Cell.

### DLS and zeta potential measurements

The hydrodynamic radius and ζ-potential of the nanoparticles were determined using a Zetasizer Ultra (Malvern Instruments Ltd, UK) in HBG at 25 °C. All measurements were performed in triplicate.

### Cell culture and cytotoxicity assay

Dose-dependent antiproliferative effects of free bPEI, SPION@bPEI, and SPION@bPEI/PIC were tested on HeLa cells (cervical cancer cell line). The antiproliferative effect of SPION@bPEI-Erb and SPION@bPEI-Erb/pGFP was tested on HeLa, MCF7 (breast cancer cell line) and HCT116 (human colon cancer cell line) cells. The cells were grown onto 75T culture flasks in Dulbecco’s Modified Eagle’s culture Medium (DMEM) with 4.5 g/L ᴅ-glucose, ʟ-glutamine, and pyruvate (Life Technologies USA) and supplemented with 10% of FBS and 1% of penicillin solution. The cells were kept at 37 °C under 5% CO_2_ and were subcultured three times per week with 0.25% trypsin/EDTA. The cells were seeded onto 96-well microtiter plates (Greiner) at a concentration of 5 × 10^3^ cells/well (HeLa and MCF7) and 10 × 10^3^ cells/well (HCT116) and incubated for 24 h at 37 °C. Then, the cells were treated with the test samples and incubated at 37 °C for 48 h. After the removal of the medium, the cells were washed with PBS several times. The antiproliferative effect of the test materials on these cell lines was evaluated by using the 3-(4,5-dimethyl-2-thiazolyl)-2,5-diphenyl-2*H*-tetrazolium bromide (MTT) cell proliferation kit (Applichem) according to the instructions from the manufacturer. In each plate, the assay was repeated for the blank medium and untreated cells in medium as controls. Then, the MTT reagent was added to each well, and the absorbance of formazan was measured at 490 nm by using a Biotek ELx800 96-well plate reader with a reference at 630 nm. The results were reported as the average of five replicates. The MTT assays were repeated at least three times and the data were presented as mean ± standard error from the mean. The statistical analysis was conducted using ANOVA and two-sample unequal variances were used to calculate the *p*-values between groups. All cell viability percentages were presented as percentages of the control viability.

### Fluorescence imaging

To investigate the in vitro optical imaging potential of the SPION@bPEI, HeLa cells were incubated in 24-well plates with free bPEI, SPION@bPEI, and SPION@bPEI/PIC at a concentration corresponding to 10 µg/mL of the bPEI content. After 2 h of incubation at 37 °C, the medium was removed and the cells were washed with PBS several times. Then, 250 µL of a 4% paraformaldehyde solution was added to each well and the plates were stored in the dark for 20 min to fix the cells. After the removal of paraformaldehyde, each well was washed three times with PBS (1 M). The fixed cell samples were examined under an Olympus-excellence RT Life Science microscope (λ_exc_ = 358 nm, λ_em_ = 485 nm).

To demonstrate the targeted delivery of nanoparticles to EGFR-overexpressing cell lines, MCF7 (EGFR-negative), HeLa (poor EGFR expressor) and HCT116 (strong EGFR expressor) cells were treated with SPION@bPEI/pGFP and SPION@bPEI-Erb/pGFP at two different pDNA concentrations (1.2 and 0.6 µg/mL) with N/P ratios of 30 and 45, respectively. The cell fixation procedure was performed 48 h after the treatment.

### Characterization

The photoluminescence spectroscopy (PL) measurements were performed by using a Horiba Jobin Yvon FluoroMax-3 spectrofluorometer at room temperature (λ_exc_ = 365 nm, λ_em_ = 482 nm for SPION@bPEI). The fluorescence microscopy images were obtained by using an Olympus-excellence RT Life Science microscope (λ_exc_ = 488 nm, λ_em_ = 510 nm for GFP).

## Results and Discussion

### Delivery of therapeutic PIC to cancer cells with SPION@bPEI and in vitro optical imaging

Highly positively charged PEI electrostatically condenses high molecular weight (MW) DNA to polypeptic nanoparticles (10–100 nm), which are capable of being absorbed by endocytosis [50–51]. Usually, the main purpose of using PEI coating on SPIONs is to provide cationic nanoparticles suitable for gene binding and transfection along with the diagnostic potential provided by the superparamagnetic iron oxide core in MRI. In this study, we focused on the label-free optical imaging potential, which is new in the literature for SPION@bPEI, and the transfection potential of these nanoparticles.

**Figure 1:**
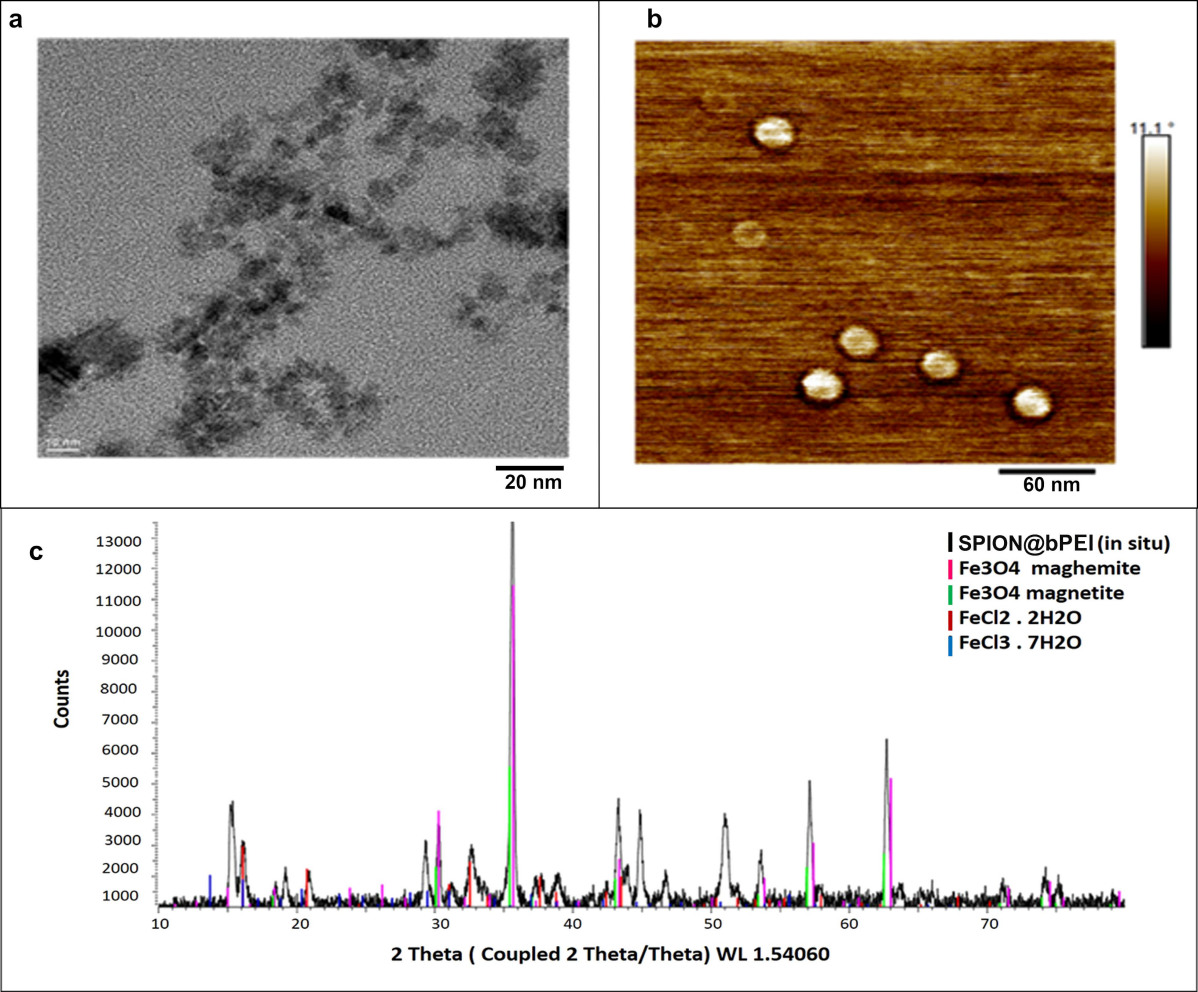
a) TEM image of SPION@bPEI. b) AFM micrograph image of SPION@bPEI (magnetic mode). c) X-ray diffraction pattern of SPION@bPEI prepared via the in situ coating method. Since the presence of the polymer prevented the observation of a diffraction pattern, it was burned under an inert atmosphere. Extra diffraction peaks are probably originating from the impurities formed as a result of the burning. Figure 1 parts b and c were reprinted from [35], O. Unal et al., “Discovery of an Exceptionally Strong Luminescence of Polyethyleneimine‐Superparamagnetic Iron Oxide Nanoparticles”, Macromolecular Chemistry and Physics, with permission from John Wiley and Sons. Copyright © 2018 WILEY-VCH Verlag GmbH & Co. KGaA, Weinheim. This content is not subject to CC BY 4.0.

A full characterization of synthesized luminescent SPION@bPEI was published by Unal et al. in 2018 [35]. The total organic content of the particles was determined to be 76% by TGA. The average crystal size of SPION@bPEI nanoparticles was measured from transmission electron microscopy (TEM) images to be approx. 6 nm (Figure 1a). The TEM images related to polymer-coated magnetic nanoparticles show that these nanoparticles seem to be agglomerated, which is due to the protruding of near particles under vacuum. It is difficult to see the PEI polymer coating around the crystal by TEM. However, it is possible to distinguish the polymer capping layer from the nanocrystal and perform image processing on a squire containing two particles protruding under vacuum using a high-resolution transmission electron microscope. For this purpose, a reduced fast Fourier transform (FFT) of a region containing two SPION@bPEI crystal particles protrude over vacuum should be generated to obtain the spatial frequency distribution in the image. Then, a noise-filtering mask from the reduced FFT preserving only the crystalline contributions from the original image should be generated to produce the filtered nanoparticle image [35].

An AFM analysis performed at magnetic mode indicated particles of approx. 20 nm in size, which suggests a slight particle aggregation (Figure 1b). According to the literature, it is usually not uncommon to obtain different results using AFM and TEM analysis. However, due to a higher resolution and material-related sensitivity the results obtained from TEM are usually more reliable [52]. The XRD pattern of SPION@bPEI synthesized in situ indicates a crystalline magnetite structure composed of both magnetite (Fe_3_O_4_) and maghemite (Fe_2_O_3_), including nanoparticles (Figure 1c). Considering only XRD patterns it would be difficult to distinguish the percentage of magnetite and maghemite in magnetic nanoparticles. However, electron paramagnetic resonance (EPR) spectroscopy analysis can be applied to overcome this problem. According to EPR spectroscopy results, SPION@bPEI nanoparticles synthesized in situ were composed of 23% magnetite and 77% of maghemite SPIONs [35].

**Figure 2:**
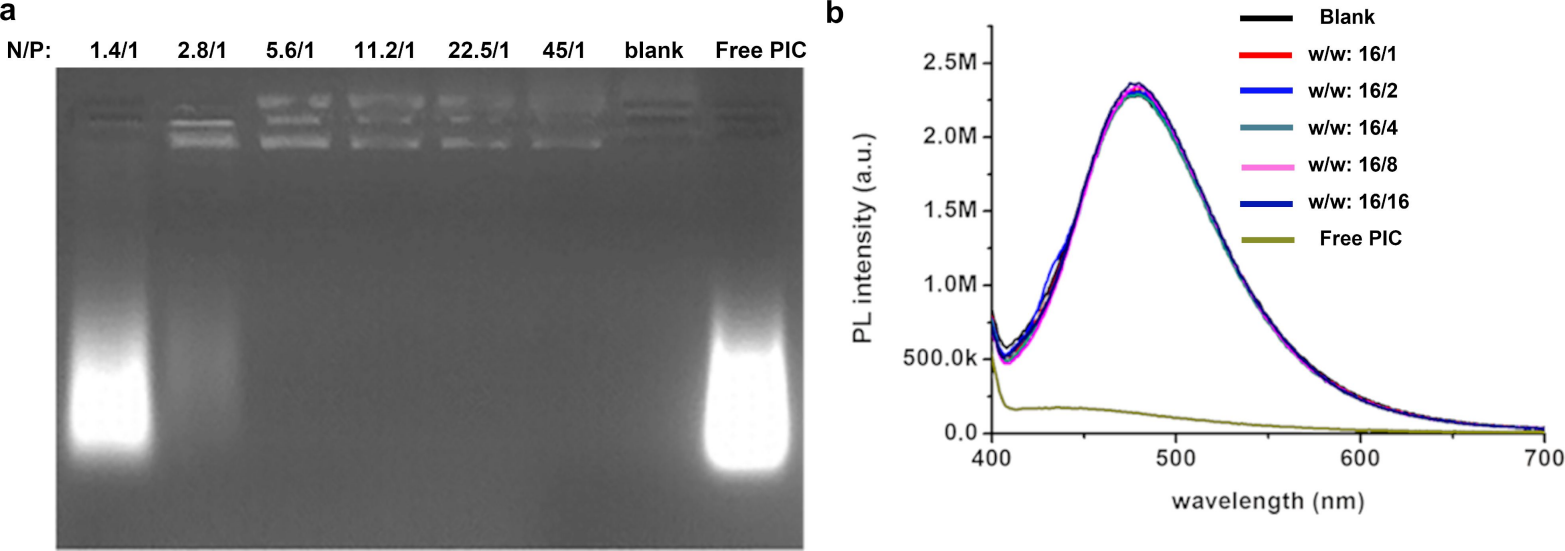
a) Gel electrophoresis showing the binding of PIC to SPION@bPEI (the PIC amount was kept constant at 4 µg/well) at different N/P ratios. b) PL spectra of free PIC, SPION@bPEI, and SPION@bPEI/PIC (1.28 mg/mL constant nanoparticle concentration) at different SPION@bPEI to PIC w/w ratios. λ_exc_ = 355 nm.

Here, the cytotoxicity of SPION@bPEI, its potential for therapeutic gene delivery, and label-free optical imaging were investigated. For this purpose, PIC which is a synthetic dsRNA was electrostatically loaded into SPION@bPEI at different N/P ratios (1.4/1, 2.8/1, 5.6/1, 11.2/1, 22.4/1, and 45/1) which corresponds to SPION@bPEI to PIC w/w ratios of 0.5/1, 1/1, 2/1, 4/1, 8/1, and 16/1. In all these compositions, the amount of PIC was kept constant at 80 µg/mL (4 µg/well). The mechanism of loading of poly I:C on nanoparticles is through electrostatic interactions between the negative charge (due to the phosphate group of dsRNA/poly I:C) and the positive charge of the nanoparticles (due to primary amine groups of polyethyleneimine). This is due to the fact that the highly positively charged PEI electrostatically condenses high molecular weight DNA or RNA to polypeptic nanoparticles (10–100 nm), which are capable of being absorbed by endocytosis [50–51]. The gel retardation of poly I:C (dsRNA analogue) happens after interacting with positively charged nanoparticles. According to our gel electrophoresis results and comparing between free poly I:C (well 8), at an N/P ratio of 1.4/1, 50% and at an N/P ratio of 2.8/1, almost all of poly I:C interacted with the nanoparticles, which resulted in the retardation of this dsRNA analogue (Figure 2a).

**Figure 3:**
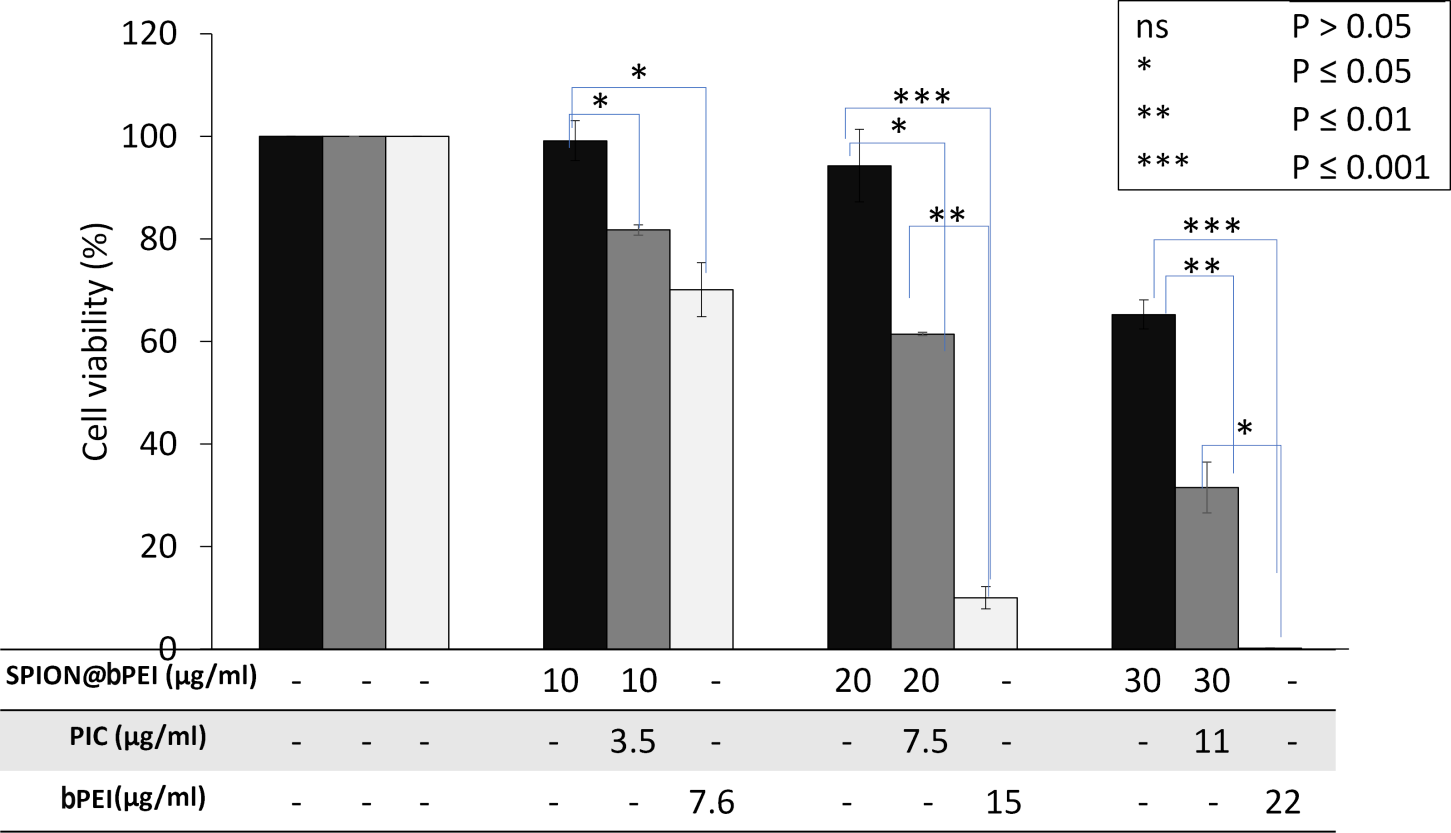
MTT cytotoxicity assay in HeLa cells treated with free bPEI, SPION@bPEI, and SPION@bPEI/PIC for 48 h (the doses are given in μg). The amount of bPEI, PIC, and SPION@bPEI given under each column identifies the amount of each component in the formulation given to the cells. The results are expressed as the mean of three independent experiments ± standard error and subjected to one-way ANOVA with Tukey's test.

The examination of the photoluminescence spectra of PIC-loaded SPION@bPEI (the amount of nanoparticles was kept constant as 1.28 mg/mL) at different SPION@bPEI to PIC w/w ratios (free PIC, 16/16, 16/8, 16/4, 16/2, 16/1, blank nanoparticles) demonstrated that PIC loading does not change the photoluminescence intensity of the SPION@bPEI nanoparticles, which is highly desirable for the theranostic function of these nanoparticles (Figure 2b). All these nanoparticles exhibit a strong blue emission with a maximum value at 480 nm when excited at 360 nm. In order to evaluate the cytocompatibility of SPION@bPEI as a gene delivery vehicle and determine the therapeutic outcome of PIC delivery by SPION@bPEI to HeLa cells, the MTT assay was used (Figure 3). Since the potentially toxic component is bPEI, the doses of the nanoparticles were reported based on their bPEI content (7.6, 15, and 22 μg/mL) determined by TGA. The HeLa cells were also treated with free bPEI for comparison. Free bPEI demonstrated a dose-dependent cytotoxicity with approx. 30% reduction in viability even at the lowest dose (7.6 μg/mL). At a 15 μg/mL dose of free bPEI, the viability was only 10%. However, when the same amounts of bPEI were introduced in the form of SPION@bPEI, no significant reduction in the viability of HeLa cells was observed at 7.6 and 15 μg/mL of bPEI (corresponding to nanoparticle concentration values of 10 and 20 μg/mL, respectively).

A significant toxicity was observed at a dose of 22 μg/mL of free bPEI, which is equivalent to a 30 μg/mL SPION@bPEI dose. This is a significant enhancement in cytocompatibility of SPION@bPEI, which may be partially due to a decreased overall positive charge upon adsorption of some amine groups to the crystal surface [53] and maybe partially due to the oxidation of amine groups as we have previously determined for SPION@bPEI [35,54]. Indeed, this is quite a notable advancement since there is no PEG on these nanoparticles to reduce the toxicity of bPEI, which is the commonly accepted method to render such toxic materials more biocompatible.

The delivery of PIC to HeLa cells with this highly cytocompatible delivery vehicle resulted in a dose-dependent viability, indicating an effective delivery of PIC into the cells at an N/P ratio of 30. The cell viability decreased by 20–40–70% with 0.9, 1.8 and 2.7 µg of PIC, respectively, delivered to the cells with an increasing dose of the SPION@bPEI/PIC. Especially at 10 and 20 µg of nanoparticles/mL dose, all the toxicity is originated from the PIC delivered into the cells, which is excellent.

**Figure 4:**
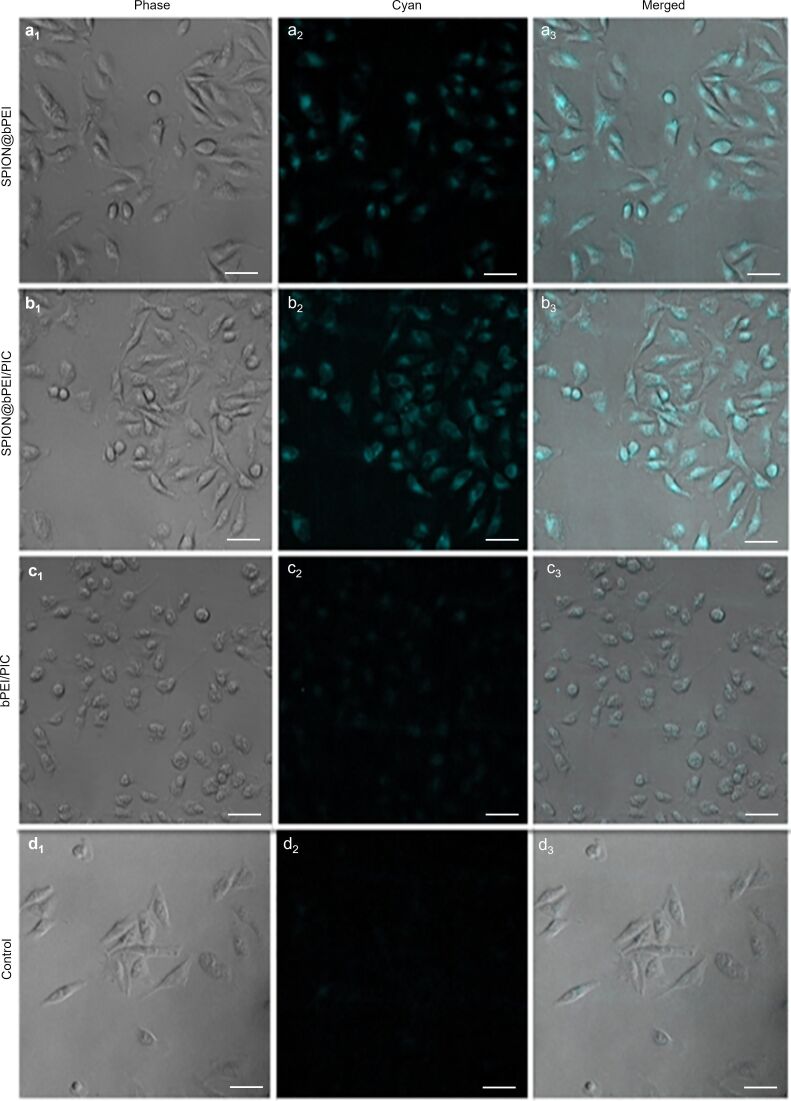
Fluorescence microscopy images of HeLa cells treated for 4 h with SPION@bPEI (20 μg/mL) (a_1_–a_3_), SPION@bPEI/PIC (N/P = 30) (b_1_–b_3_), free bPEI (15 μg/mL) (c_1_–c_3_), and control untreated cells (d_1_–d_3_) at a 20× magnification (λ_ex_ = 358 nm, λ_em_ = 480 nm). Images subscribed as 1 are the phase-contrast microscopy images, images subscribed as 2 are fluorescence microscopy images (cyan), and images subscribed as 3 are merged images. The scale bar for the images is 50 µm.

One of the most attractive features of this SPION@bPEI is the intense blue emission, which makes additional fluorescent tagging unnecessary. Fluorescence microscopy images of the cells treated with SPION@bPEI show a strong intracellular optical signal, proving these particles as good optical imaging agents (Figure 4a_1_–a_3_). SPION@bPEI/PIC also displayed a similar, strong blue luminescence in the cytoplasm of the cells, allowing for the optical tracking of these nanoparticles (Figure 4b_1_–b_3_). Such strong intracellular signal of SPION@bPEI/PIC is in agreement with the spectroscopic data (Figure 2b). Cells treated with free bPEI show a very weak optical signal in the cytoplasm, as expected (Figure 4c_1_–c_3_). In addition, significant morphological changes were observed in these cells due to the cytotoxic effect of bPEI on the cytoplasmic membrane and the triggering of an intrinsic apoptotic pathway [55–57]. Most probably, the weak luminescence and high toxicity at the required concentrations to observe the fluorescence signal are the reasons why bPEI luminescence has not been detected by fluorescence microscopy studies before [18,58].

### Targeted delivery of GFP to EGFR-positive cells with luminescent SPION@bPEI

After the demonstration of the theranostic potential of SPION@bPEI, these nanoparticles were tagged with Erb for selective delivery of the cargo to EGFR-overexpressing tumor cells, as an example of receptor-mediated targeting of these nanoparticles. According to our calculation, 12.5 mg/mL of SPION@bPEI-Erb nanoparticles contains 380 µg of Erb/mL, which was determined from the unbound Erb using the Bradford assay (section “PIC and pGFP loading to luminescent magnetic nanoparticles (SPION@bPEI/PIC or SPION@bPEI/pGFP)”). Therefore, SPION@bPEI-Erb nanoparticles contain about 3% of Erb (w/w).

The average hydrodynamic sizes of SPION@bPEI and SPION@bPEI-Erb were measured to be 69 nm and 7.5 nm in HBG, respectively (Supporting Information File 1, Figure S1). Due to the high volume-to-surface-area ratio, SPIONs tend to attract each other and aggregate to minimize their high surface energies [59]. Therefore, electrostatic and steric repulsion need to be created between SPIONs to prevent agglomeration and produce a stable nanoparticle. It was also demonstrated that peptization prevents agglomeration, paving the way for the production of stabilized magnetic nanoparticles [60]. The antibody conjugation may stabilize the particles further, preventing aggregation and hence reducing the hydrodynamic size [61]. Therefore, Erb conjugation may result in a reduction of the surface energy of magnetic nanoparticles and pave the way for a decrease in the hydrodynamic radius by preventing agglomeration and increasing stabilization of the nanoparticles.

The zeta potential values of SPION@bPEI and SPION@bPEI-Erb were measured to be +35.2 mV and +29.1 mV, respectively (Supporting Information File 1, Table S1), which is expected since the conjugation of Erb to nanoparticles consumed some amines, and the antibody contains anionic functionalities as well. The Erb conjugation decreased the luminescence intensity of SPION@bPEI by approx. 30%, but PIC loading (Figure 2b) or DNA loading did not change the luminescence intensity any further (Supporting Information File 1, Figure S2).

**Figure 5:**
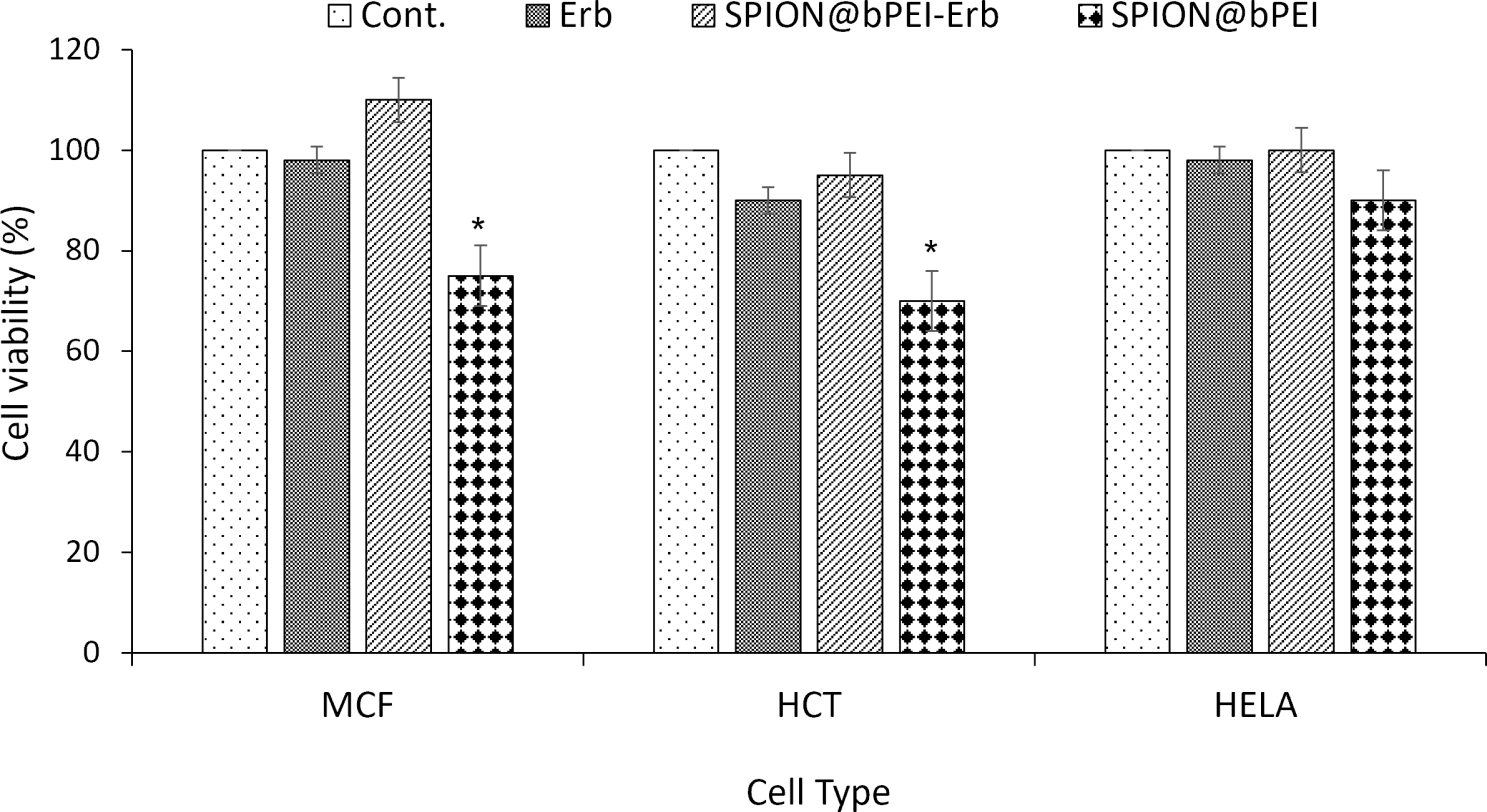
MTT cytotoxicity assay in MCF7, HCT116, and HeLa cells treated with free Erb (0.62 μg/mL), SPION@bPEI (19.2 μg/mL), and SPION@bPEI-Erb (20.7 μg/mL). The results are expressed as the mean of three independent experiments ± standard error and subjected to one-way ANOVA with Tukey's test.

The cytotoxicity of Erb-conjugated SPION@bPEI nanoparticles (SPION@bPEI-Erb) and free Erb at an equivalent concentration to the Erb content of SPION@bPEI-Erb were investigated in MCF7, HeLa, and HCT116 cell lines (Figure 5) at a single dose. SPION@bPEI seems to be more toxic to MCF7 and HCT116 than to HeLa cells at the tested dose of 19.2 µg/mL. Such cell line-based differences are normal since the mechanism of nanoparticle internalization may vary depending on the cell type, cell cycle stage, and cell polarization state [62].

Free Erb did not cause any significant cytotoxicity at the applied dose (0.62 μg/mL) in either of the cell lines studied, which was desired in this study since Erb was only used to demonstrate molecular targeting. Although HCT116 cells are EGFR positive, the IC_50_ of the Erb monoclonal antibody is approx. 380 μg/mL [63] and, hence, no significant Erb-dependent cytotoxicity was expected. Interestingly, Erb conjugation significantly reduced nanoparticle cytotoxicity. This may be due to the reduction of surface amine groups, which is the main reason for the cytotoxic effect of PEI-coated nanoparticles. It is also possible that the Erb modification may change the internalization pathway of the nanoparticles. As an example, in 2009, Gabrielson et al. demonstrated that folic acid modification of PEI polyplex enhances internalization via a caveolar pathway in cells expressing folate receptors [64]. Since caveolae-mediated internalization pathways do not necessarily involve traffic to lysosomes, it can yield a better transfection efficiency [65]. Indeed, it was shown that both unmodified branched and linear PEI polyplexes could damage plasma membranes, resulting in a rapid redistribution of phosphatidylserine from the inner plasma membrane to the outer cell surface (without activation of caspase 3) and also leakage of cytosolic lactate dehydrogenase (LDH) from the cells [55–56]. It has also been known that unmodified polyplexes can switch on the intrinsic apoptotic pathway by activating the box protein and therefore releasing the cytochrome c [57]. Covalent binding of Erb to the surface of SPION@bPEI may somehow switch off the intrinsic cytotoxicity by hindering the primary amine interaction with box proteins. These can be some of the possible reasons for cytotoxicity reduction of SPION@bPEI after surface modification with Erb.

**Figure 6:**
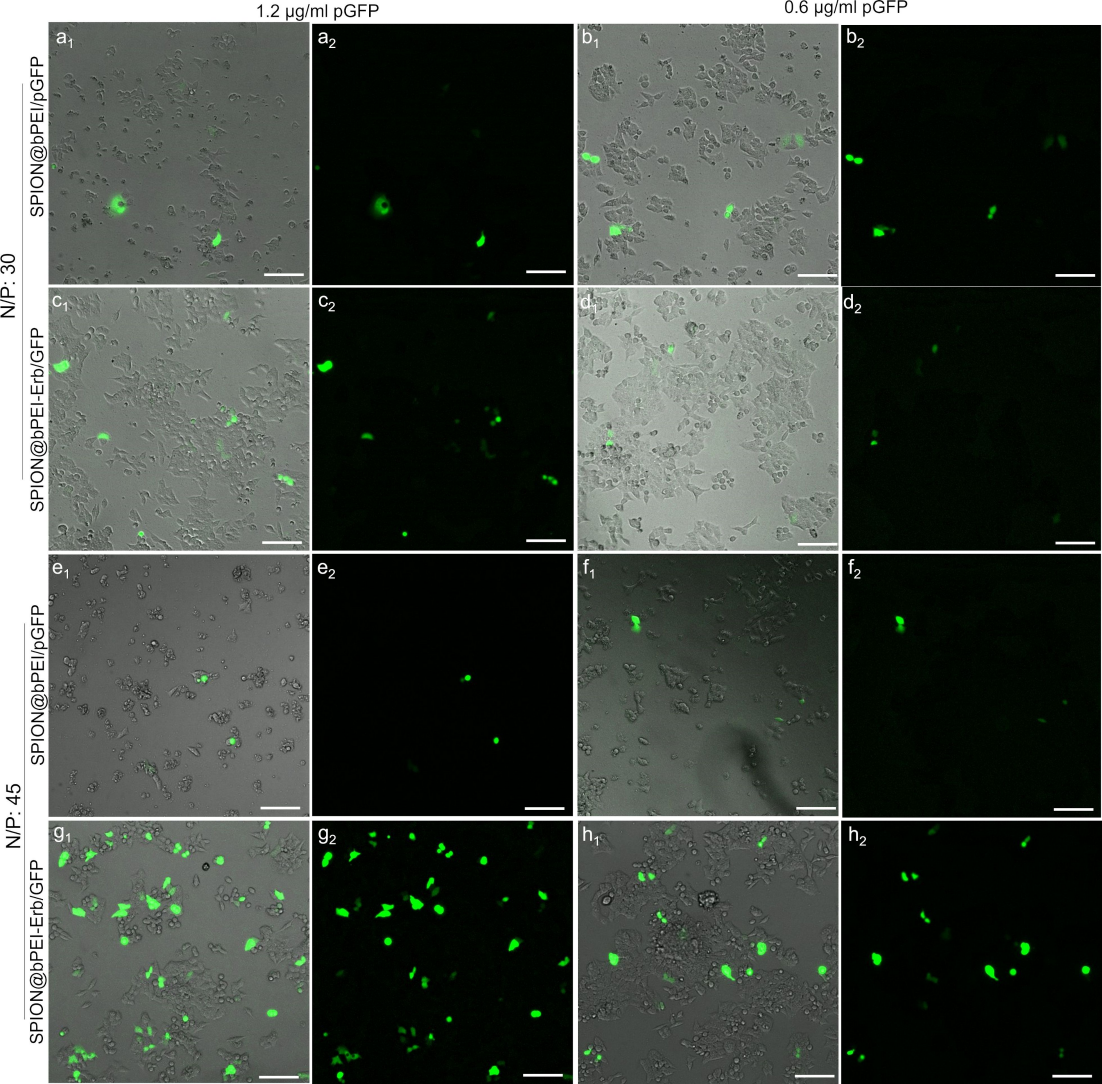
Fluorescence microscopy images of HCT116 cells transfected with SPION@bPEI/pGFP or SPION@bPEI-Erb/pGFP at an N/P ratio of 30 (5a–d) with a) 1.2 µg/mL pDNA loaded on 12.8 µg/mL SPION@bPEI, b) 0.6 µg/mL pDNA loaded on 6.4 µg/mL SPION@bPEI, c) 1.2 µg/mL pDNA loaded on 13.8 µg/mL SPION@bPEI-Erb, d) 0.6 µg/mL pDNA loaded on 6.9 µg/mL SPION@bPEI-Erb. Fluorescence microscopy images of HCT116 cells transfected with SPION@bPEI/pGFP or SPION@bPEI-Erb/pGFP at an N/P ratio of 45 (5e–h) with e) 1.2 µg/mL pDNA loaded on 19.2 µg/mL SPION@bPEI, f) 0.6 µg/mL pDNA loaded on 9.6 µg/mL SPION@bPEI, g) 1.2 µg/mL pDNA loaded on 20.7 µg/mL SPION@bPEI-Erb, h) 0.6 µg/mL pDNA loaded on 10.35 µg/mL SPION@bPEI-Erb. All images were taken 48 h after transfection. Images with subscription 1 are merges images, and images with subscription 2 are green fluorescence microscopy images. Scale bar = 100 µm.

To empirically optimize the transfection efficiency and find a safe dose, the GFP plasmid was loaded on SPION@bPEI and SPION@bPEI-Erb at two N/P ratios (30 and 45) and tested on EGFR-positive HCT116 cells in two different DNA concentrations: 1.2 and 0.6 μg/mL (Table 1, Figure 6). Since the primary goal here was to demonstrate Erb specific internalization of nanoparticles by the target cells, both Erb and the cargo were used in nontoxic concentrations.

**Table 1:** Composition of the particles used for pGFP transfection.

	SPION@bPEI				SPION@bPEI-Erb			
		
N/P ratio	30		45		30		45	

NP (μg/mL)	12.8	6.4	19.2	9.6	13.8	6.9	20.7	10.35
pGFP	1.2	0.6	1.2	0.6	1.2	0.6	1.2	0.6
Erb (μg/mL)	–	–	–	–	0.42	0.21	0.62	0.31

**Figure 7:**
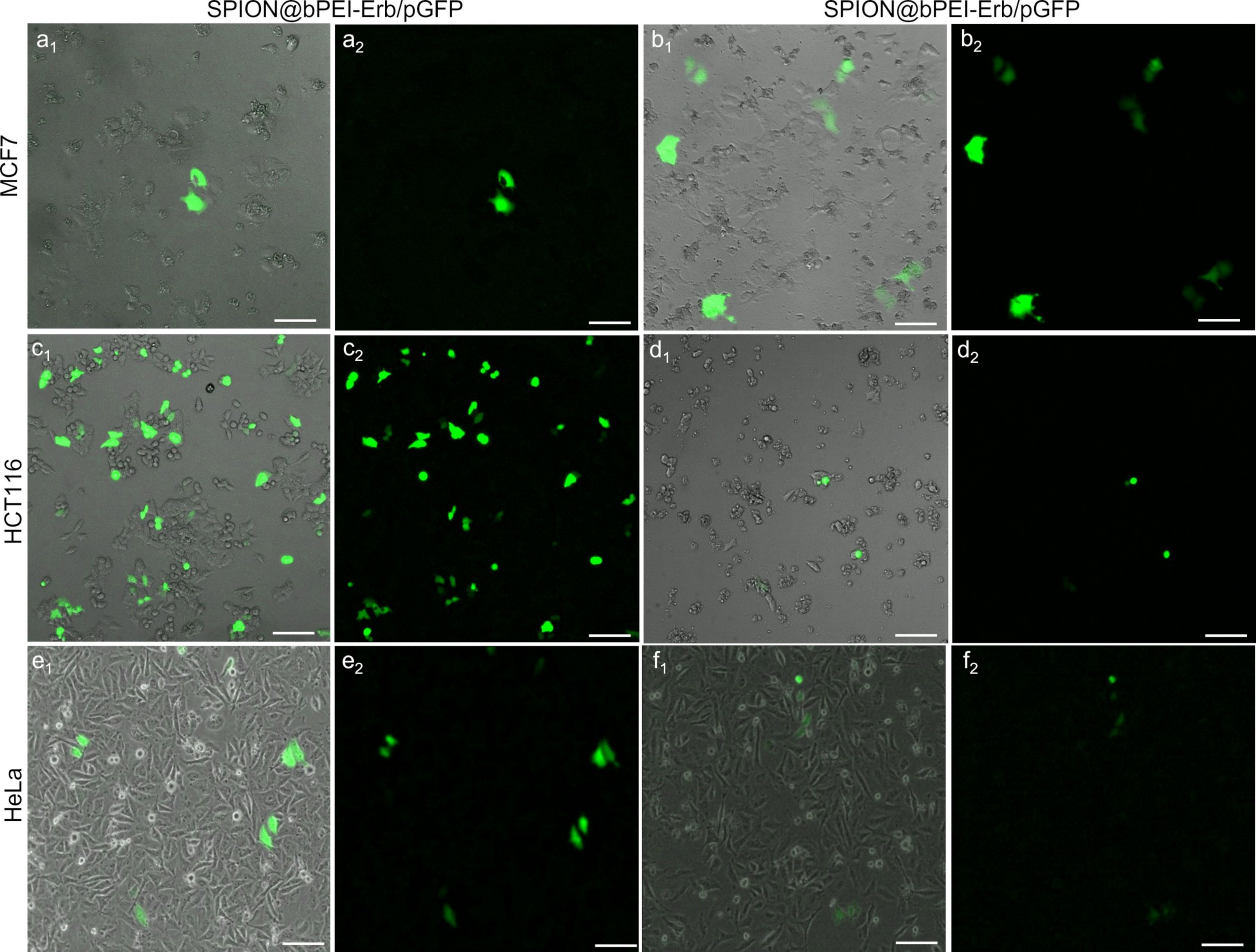
Fluorescence microscopy images of MCF7, HCT116, and HeLa cells transfected with SPION@bPEI-Erb/pGFP or SPION@bPEI/pGFP at N/P ratio of 45, 48 h after transfection: a-c-e: 1.2 µg/mL pDNA loaded on 20.7 µg/mL SPION@bPEI-Erb; b-d-f: 1.2 µg/mL of pDNA loaded on 19.2 µg/mL SPION@bPEI. Images with subscribed as 1 are the merged images, and images subscribed as 2 are green fluorescence microscopy images. Scale bar = 100 µm.

Fluorescence microscopy images of nanoparticle treated cells indicate that the transfection with SPION@bPEI was not efficient at either dose or N/P ratios. However, Erb-conjugated nanoparticles transfected the EGFR positive HCT116 cells very efficiently at an N/P ratio of 45. Besides, SPION@bPEI/pGFP induced significant toxic effect on HCT116 cells even at 12.8 µg/mL. However, SPION@bPEI-Erb/pGFP nanoparticles did not induce any cytotoxic effect even at 20.7 µg/mL. This is in agreement with the MTT assay results (Figure 5). The optimized formulation and dose were also applied to HeLa and MCF7 cell lines under identical conditions. Erb-tagged nanoparticles enhanced GFP transfection to HeLa cells but not as much as the transfection observed in HCT116, as expected. HeLa cells are known to have a very low level of EGFR expression [66]. Hence, Erb tagging slightly enhanced particle uptake and GFP transfection compared to untagged nanoparticles. As expected, Erb tagging did not improve the transfection efficiency of EGFR-negative MCF7 cells, confirming the selective delivery of the nanoparticles. Microscopy images also indicate that Erb conjugation not only increases the transfection efficiency but also decreases the toxicity of SPION@bPEI on HCT116 and HeLa cells (Figure 7), which is in agreement with the cytotoxicity results.

## Conclusion

In this study, bPEI-coated cationic superparamagnetic iron oxide nanoparticles with a very strong intrinsic blue luminescence were demonstrated as promising theranostic nanoparticles. The optical imaging and diagnostic potential of these nanoparticles were confirmed by a strong intracellular blue luminescence observed with a fluorescence microscope. Considering the strong T2 signal of SPION@bPEI [35], these nanoparticles may be considered as dye-free dual-mode (MRI and optic) imaging agents. Such combinations are highly desired for diagnostics combined with intraoperative imaging, ex vivo analysis of nanoparticle distribution, among others.

These SPION@bPEI nanoparticles are significantly more cytocompatible than bPEI despite their cationic nature. The binding of bPEI to the crystal surface and partial amine oxidation are suggested as possible reasons of the enhanced cytocompatibility. Yet, bPEI coating is still capable of delivering oligonucleotides as proven here by two different cargoes. Poly I:C, a pro-apoptotic agent, was loaded on these nanoparticles (N/P = 30), and was effectively delivered to HeLa cells, reducing the viability by 40% in 48 h at a nontoxic dose of the nanoparticle. Besides, PIC loading did not interfere with the luminescence properties of the nanoparticles, hence it provided the ability to visualize the internalization of the gene-delivering nanoparticles by the cells via optical imaging.

SPION@bPEI nanoparticles were also tagged with Erb to deliver an anionic cargo to EGFR-overexpressing cells as a demonstration of selective targeting. SPION@bPEI-Erb was loaded with nontoxic, widely used cargo: pGFP. A significantly enhanced GFP expression in EGFR-positive HCT116 cells and a slightly improved transfection in low EGFR expressing HeLa, but not in EGFR negative MCF7 cells, proved the targeting potential of these transfection vehicles [67–69]. The applied Erb concentration to modify nanoparticle surfaces was almost 1% of the toxic doses [63]. Therefore, selective internalization of the target cells with no toxicity was achieved. This result, coupled with the biocompatibility of the nanoparticles compared to bPEI and the small hydrodynamic size make these nanoparticles quite promising for future in vivo targeted gene delivery studies coupled with optical imaging. Lastly, SPION@bPEI was slightly toxic to MCF7 and HCT116 cell lines. The cytotoxicity was eliminated after Erb conjugation, possibly due to altered cell internalization mechanisms.

Overall, the combination of SPIONs, which are the only FDA approved nanoparticles for imaging, and bPEI, which is the golden standard for non-viral gene transfection with an added advantage of strong luminescence in this unique SPION@bPEI structure, holds great potential for effective gene/drug delivery coupled with dual-mode imaging.

## Supporting Information

File 1 Supporting Information contains dynamic light scattering (DLS), photoluminescence spectra, and zeta potential analysis of SPION@bPEI, SPION@bPEI/pDNA, SPION@bPEI-Erb, SPION @bPEI-Erb/pDNA, and SPION@bPEI-Erb/pDNA.
