# Supplementary material for: Theranostic potential of self-luminescent branched polyethyleneimine-coated superparamagnetic iron oxide nanoparticles
Source: Beilstein J Nanotechnol. 2022 Jan 18;13:82–95. doi: 10.3762/bjnano.13.6 (PMC8787352; doi:10.3762/bjnano.13.6)
Supplement: File 1 — Supporting Information contains dynamic light scattering (DLS), photoluminescence spectra, and zeta potential analysis of SPION@bPEI, SPION@bPEI/pDNA, SPION@bPEI-Erb, SPION @bPEI-Erb/pDNA, and SPION@bPEI-Erb/pDNA. [file Beilstein_J_Nanotechnol-13-82-s001.pdf]

BEILSTEIN JOURNAL OF NANOTECHNOLOGY

# Supporting Information

for

# Theranostic potential of self-luminescent branched polyethyleneimine-coated superparamagnetic iron oxide nanoparticles

Rouhollah Khodadust, Ozlem Unal and Havva Yagci Acar

*Beilstein J. Nanotechnol.* **2022,** *13,* 82–95. doi:10.3762/bjnano.13.6

## Supporting Information contains dynamic light scattering (DLS), photoluminescence spectra, and zeta potential analysis of SPION@bPEI, SPION@bPEI/pDNA, SPION@bPEI-Erb, SPION @bPEI-Erb/pDNA, and SPION@bPEI-Erb/pDNA

License and Terms: This is a supporting information file under the terms of the Creative Commons Attribution License (https://creativecommons.org/licenses/by/4.0). Please note that the reuse, redistribution and reproduction in particular requires that the author(s) and source are credited and that individual graphics may be subject to special legal provisions.
The license is subject to the *Beilstein Journal of Nanotechnology* terms and conditions: (https://www.beilstein-journals.org/bjnano/terms)

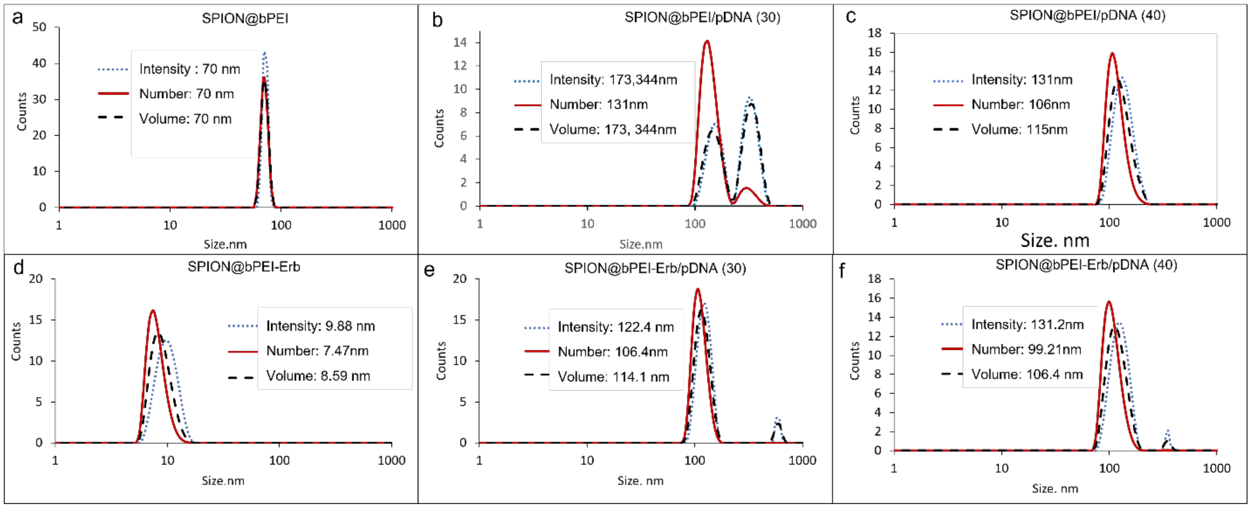


**Figure S1:** Panels (a–f) demonstrate the size of SPION@bPEI, SPION@bPEI/pDNA(30), SPION@bPEI/pDNA(40), SPION@bPEI-Erb, SPION @bPEI-Erb/pDNA(30), and SPION@bPEI-Erb/pDNA(40) complex, respectively. The size of nanoparticles is represented in all three forms as intensity, number, and volume. The numbers (30) and (40) represent the N/P ratios.

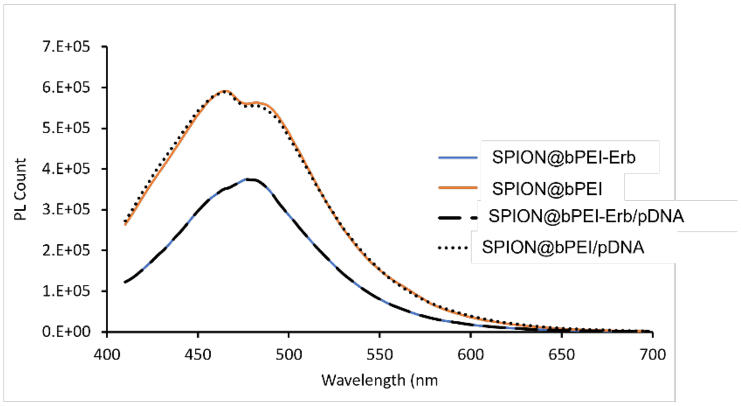


**Figure S2:** Photoluminescence spectra of SPION@bPEI and SPION@bPEI-Erb before and after plasmid DNA loading (SPION@bPEI/pDNA and SPION@bPEI-Erb/pDNA).

**Table S1:** Zeta potential of SPION@bPEI, SPION@bPEI/pDNA(30), SPION@bPEI/pDNA(40), SPION@bPEI-Erb, SPION@bPEI-Erb/pDNA(30), and SPION@bPEI-Erb/pDNA(40) complex.

| Sample | Zeta Potential Values (mV) |
|---|---|
| SPION@bPEI | 35.2 |
| SPION@bPEI/pDNA (N/P=30) | 23.62 |
| SPION@PEI/pDNA (N/P=40) | 27.68 |
| SPION@bPEI-Erb | 29.1 |
| SPION@bPEI-Erb/pDNA(N/P=30) | 21.6 |
| SPION@bPEI-Erb/pDNA(N/P=40) | 25.89 |
